# ImaGene: a convolutional neural network to quantify natural selection from genomic data

**DOI:** 10.1186/s12859-019-2927-x

**Published:** 2019-11-22

**Authors:** Luis Torada, Lucrezia Lorenzon, Alice Beddis, Ulas Isildak, Linda Pattini, Sara Mathieson, Matteo Fumagalli

**Affiliations:** 1 Department of Life Sciences, Silwood Park campus, Imperial College London, Buckhurst Road, Ascot, SL5 7PY UK; 2 Department of Electronics, Information and Bioengineering, Politecnico di Milano, piazza Leonardo da Vinci 32, Milan, 20133 Italy; 3 Department of Biological Sciences, Middle East Technical University, METU Üniversiteler Mah. Dumlupınar Blv. No:1, Ankara, 06800 Çankaya Turkey; 4 Department of Computer Science, Swarthmore College, 500 College Ave, Swarthmore, 19081 PA USA

**Keywords:** Population genetics, Natural selection, Supervised machine learning, Convolutional neural networks

## Abstract

**Background:**

The genetic bases of many complex phenotypes are still largely unknown, mostly due to the polygenic nature of the traits and the small effect of each associated mutation. An alternative approach to classic association studies to determining such genetic bases is an evolutionary framework. As sites targeted by natural selection are likely to harbor important functionalities for the carrier, the identification of selection signatures in the genome has the potential to unveil the genetic mechanisms underpinning human phenotypes. Popular methods of detecting such signals rely on compressing genomic information into summary statistics, resulting in the loss of information. Furthermore, few methods are able to quantify the strength of selection. Here we explored the use of deep learning in evolutionary biology and implemented a program, called ImaGene, to apply convolutional neural networks on population genomic data for the detection and quantification of natural selection.

**Results:**

ImaGene enables genomic information from multiple individuals to be represented as abstract images. Each image is created by stacking aligned genomic data and encoding distinct alleles into separate colors. To detect and quantify signatures of positive selection, ImaGene implements a convolutional neural network which is trained using simulations. We show how the method implemented in ImaGene can be affected by data manipulation and learning strategies. In particular, we show how sorting images by row and column leads to accurate predictions. We also demonstrate how the misspecification of the correct demographic model for producing training data can influence the quantification of positive selection. We finally illustrate an approach to estimate the selection coefficient, a continuous variable, using multiclass classification techniques.

**Conclusions:**

While the use of deep learning in evolutionary genomics is in its infancy, here we demonstrated its potential to detect informative patterns from large-scale genomic data. We implemented methods to process genomic data for deep learning in a user-friendly program called ImaGene. The joint inference of the evolutionary history of mutations and their functional impact will facilitate mapping studies and provide novel insights into the molecular mechanisms associated with human phenotypes.

**Electronic supplementary material:**

The online version of this article (10.1186/s12859-019-2927-x) contains supplementary material, which is available to authorized users.

## Background

The quest for a deeper understanding of the molecular mechanisms underpinning phenotypic variation has transformed population genetics into a data-driven discipline. Thanks to the technological advances in RNA/DNA sequencing [1] coupled with exponentially increasing computational power, we are now in the position to process large amounts of genomic data to address some still unanswered questions in this field.

By far, one of the most elusive questions in evolutionary biology is to what extent adaptation has shaped the genomes of extant species. The identification of signatures of natural selection in the genome has the importance of (i) assessing the ability of endangered species to respond to climate change [2] and (ii) identifying functional variants underlying notable or disease-related phenotypes [3]. In fact, genetic variants that are characteristic of past natural selection in the human genome have frequently been linked with a wide spectrum of phenotypes of medical relevance [4, 5].

A large range of methods for detecting genomic signatures of natural selection from sequencing data have been proposed [6]. Most of the efforts have been devoted towards the identification of positive selection, the situation whereby beneficial mutations that confer an increased fitness to the carrier become more common in a population. Positive selection is the main driver of genetic adaptation for many species, including humans [7]. Current methods to detect natural selection are largely based on compressing the information about population genomic variation into summary statistics, whose distribution under neutrality can be empirically or analytically derived [8]. For instance, summary statistics may carry information about the locus-specific distribution of allele frequencies [9] or haplotype structure [10, 11].

However, most adaptive processes happened via weak-to-moderate selection from standing variation [12]. As such, a large proportion of selective events have left genomic signatures which are cryptic, complex and hard to detect when employing only a limited number of summary statistics. To overcome this dilemma, likelihood-free methods have been successfully applied to detect selection signatures, via approximate Bayesian computation [13], unsupervised [14] or supervised machine learning (ML) [15–17]. In contrast to classic modelling, ML algorithms maximize the predictive accuracy by automatically and iteratively tuning their internal parameters while remaining relatively unconscious of the phenomenon they are trying to predict. While unsupervised methods attempt to learn the underlying structure in the data without knowledge of the ground truth, supervised ML algorithms require the specification of a known data set, called a training set, to make predictions on new unknown data sets [18].

A recently reintroduced class of supervised ML algorithms is deep learning [19], an inference framework based on artificial neural networks (ANN). ANNs comprise inputs (also called features) and outputs (responses), connected by nodes in a series of hidden layers [20]. Connections between nodes are optimized using the training set to minimize the predictive error. After training, an ANN can predict the response given any arbitrary new data it receives in entry. Deep learning algorithms are now heavily applied in biology [21] and genomics to predict, for instance, protein binding sites, splice junctions or compound-protein interactions [22]. Whilst promising, their use in evolutionary genomics is still relatively new [23].

Despite their ability to handle many correlated features, the most established deep learning algorithms used in evolutionary genomics still rely on reducing the information into summary statistics [24, 25]. Summary statistics are typically calculated to reduce data dimensionality while capturing most of the relevant information. An alternative approach makes full use of genomic information and processes population genomic data by image representation. For instance, at the intra-population level, rows may correspond to individual sampled haplotypes, columns represent the genomic location of each locus, and each pixel’s color is a discrete value defining the occurrence of a specific allele [26]. Such image representations of population genomic data can be directly used to infer selective events [27, 28]. In fact, this data representation maintains the original information and allows the use of algorithms for image processing. Therefore, the detection of selection signatures in the genome directly translates into a problem of pattern recognition in image analysis.

Under such data representation, Convolutional Neural Networks (CNNs) are the most suitable class of algorithms for feature extraction and prediction, as CNNs are a branch of ANNs specifically designed for processing images. As each pixel would be considered a unique feature, standard ANNs would be unnecessarily complex. Instead, CNNs use several layers of filtering (called convolution), each one processing adjacent pixels grouped in windows, which are then moved to cover the whole image [29]. Weights associated with each filter are then iteratively adjusted during the training to detect informative local patterns. Therefore, convolution layers serve the additional purpose of automatically extracting informative features which are then passed as input units to several fully connected layers for the prediction.

CNNs have been recently applied to population genomic data to infer recombination hotspots [30] and various population genetic parameters [25, 28]. Given the inevitable lack of real data, training data was generated via simulations conditional on a known demographic model. While such pioneering studies show how promising deep learning algorithms are in the field of population genomics, there are still several open questions in the use of CNNs in evolutionary biology for characterizing natural selection.

First, all current implementations aim at classifying regions into neutrally evolving or targeted by either soft or hard sweep [28] without estimating any parameter of the event (e.g. timing or strength). Also, a comprehensive assessment of how population genomic data should be presented as input to a CNN is still missing. Finally, it is not clear whether such algorithms are scalable for processing large sample sizes and whole-genome data, and whether they are sufficiently robust to model assumptions used to generate the training data.

In this study, we aim to address these shortcomings and implement a CNN-based approach to detect and quantify natural selection from population genomic data. After providing an overview of several possible image representations of population genomic data, we assess how both data manipulation and model specification can affect the accuracy for detecting and quantifying natural selection. We implement this inference in a user-friendly open source program, ImaGene, available at https://github.com/mfumagalli/ImaGene.

## Implementation

The image representation of population genomic data is suitable to translating pattern recognition algorithms for the inference of evolutionary parameters, as recently proposed [28, 30]. Here, we took advantage of this observation and implemented a CNN-based scalable classification pipeline in *python*, called ImaGene, to quantify natural selection from genomic data.

ImaGene consists of the following steps:

1. generate training and testing sets by performing simulations of population genomic data conditional on a demographic model and selection events;
2. process all simulations, convert them into images, divide them into training, validation and testing sets;
3. train and test the network using *Keras*, and output several metrics including the probability distribution for the parameter of interest.

Current interactivity consists of *python* objects to set all options for each stage of the pipeline.

As an illustration for the whole pipeline, in this manuscript we assume that our aim is to detect and quantify a positive selection event, with weak-to-moderate magnitude, that occurred 15,000 years ago in a European human population with an initial population allele frequency of 1%.

### Step 1: simulations

In ImaGene, the training set is built via simulations using *msms* [31]. For the illustrative purpose outlined above, we assume a plausible demographic model describing the history of a European population [32]. The user also decides the range for the selection coefficient to be estimated and any other parameter of interest, including the sample size and the number of simulations.

In this manuscript, as an illustration, we performed more than 2 million simulations of genomic regions of 80 kbp for 128 chromosomes representing unrelated CEU individuals (CEU: Utah Residents with Northern and Western European Ancestry) in the 1000 Genomes Project [33]. We assumed a mutation rate of 1.5×10^−8^ per base per generation and a uniform recombination rate of 1.0×10^−8^ per base pairs per generation, in line with realistic values for the human genome [34, 35]. Finally, population parameters were scaled using a reference effective population size (*N*_*e*_) of 10,000.

### Step 2: image representation

Population genomic data is usually represented as letters (nucleotides A,C,G,T) arranged in strings (chromosomes) piled up in stacks (individuals or populations). An alternative approach to process population genomic data is by image representation. Images are tridimensional matrices with the third dimension being the color. In the simplest scenario, populations are arranged along the height (rows), loci along the width (columns), and the sample frequency of each allele along the depth (color). In other words, each pixel contains information about the frequency of each one of four possible nucleotides. Therefore, each vector of nucleotide frequencies encodes a specific color in the CMYK scale.

As much of human genetic variation is diallelic, it is convenient to convert such full-color images to black and white ones. The color dimension is now reduced to length one, and each pixel encodes the frequency of one of the two alleles for a population in a locus. When data from one or more outgroup species are available, it is possible to infer the ancestral state for each polymorphism. Under such circumstances it is usual to report the frequency of the derived allele (as opposed to the ancestral state). When such information is not available, the frequency of the least frequent allele (usually referred to as minor) is considered. At the intra-population level, individual sampled haplotypes (from phased genotypes) are instead ordered on rows and the color of each pixel is a discrete value out of four possibilities. Again, alleles can be transformed into binary values to produce black and white images, with a polarization based on ancestral or major states.

In practice, this step consists of several intermediate stages. Files encoding simulations from *msms* are parsed and converted to binary matrices. If the ancestral state of the locus of interest is unknown, the alignment is recoded so that the most frequent allele in each column is converted into zeros, and the least frequent allele into ones. Rows and columns can be then sorted using different criteria. For instance, they can be ordered by their number of occurrence. A filter on the minor allele frequency can be set by the user, otherwise each column is considered. No monomorphic site is recorded within the simulations nor converted into images.

Each image in the training set is required to have same dimensions. However, this condition is not guaranteed given the stochasticity of simulations which can produce a different number of polymorphic sites. In ImaGene, users can resize images to have the same dimensions. For instance, columns can be resized to the average value across the training set or to arbitrary preset values. Resized images and their corresponding labels are then shuffled randomly and split into the training, validation and testing data sets. Parameter values are converted into class labels, and these can be transformed into probability mass functions.

### Step 3: prediction and quantification

ImaGene directly interacts with *Keras* models [36] and therefore the user can define her/his own architecture and hyperparameters. ImaGene provides utilities to monitor and evaluate the training and it uses a standard approach for binary and multiclass classification tasks.

Current implementations of deep learning to estimate continuous parameters in population genetics use a final layer comprised of a regression step [24, 28]. By taking advantage of the Bayesian interpretation of class scores in ANNs/CNNs [37], ImaGene allows users to estimate continuous variables via multiclass classification of discrete intervals from the posterior distribution of the parameter of interest.

During training, the loss function is defined as the dissimilarity between the predicted distribution and the true one, and we measure it using the cross-entropy function. ImaGene allows the user to define the true distribution in different ways. An intuitive method consists of placing all the mass of the distribution on the true class resulting in a Dirac delta distribution (also called categorical), where all the other vector elements are zeros. However, this definition does not increasingly penalize dissimilarity as it happens farther from the true label. Therefore, we also implement Gaussian distributions with mean equal to the true class and variable variance. ImaGene also implements a procedure that randomly perturbs true labels to some fixed margin.

A point estimate for the parameter of interest (e.g. selection coefficient) is given by either the maximum *a posteriori* (MAP) value or the posterior mean of the probability distribution over all classes. Likewise, highest posterior density intervals (HPDI) can be obtained from the estimated posterior distribution by Monte Carlo sampling. Finally, model testing (e.g. for the presence of natural selection) can be performed by calculating Bayes factors.

## Results

### Image representations of population genomic data

**Fig. 1:**
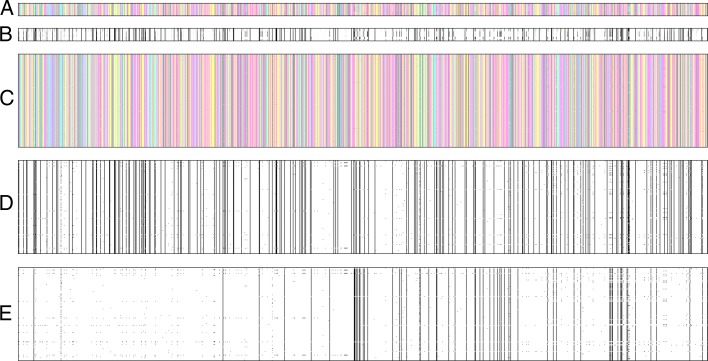
Image representations of human population genomic data for *EDAR* gene. In panels **a** and **b**, each row represents a population from the 1000 Genomes Project data set, sorted from the top to the bottom by increasing geographical distance from central Africa. Each pixel encodes for the frequency of four nucleotides (panel **a**) or the derived allele (panel **b**) for each polymorphism. Panels **c**-**e** refer to the Han Chinese population only, and each row represents a sampled haplotype. Pixel encodes for the frequency of four nucleotides (**c**), the derived allele (**d**) or the minor allele calculated across all populations (**e**)

As an illustration, we produced images from population genomic data for a notable human gene of interest, *EDAR* (Fig. 1). This gene contains alleles associated with multiple phenotypes in several human populations [38, 39], and it is a well-known target of positive selection in East Asians [10, 40]. In Fig. 1a-b, each row represents a human population from the 1000 Genomes Project [33] sorted from top to bottom by their geographical distance from central Africa. For ease of visualization, only loci which are polymorphic in at least one population (i.e. at least one heterozygote is observed) are reported.

A visual inspection of Fig. 1a-b reveals a pattern of horizontal clustering and differentiation between populations. In particular, rows representing populations in East Asia appear to be highly homogeneous within themselves but largely deviating from others. This is in line with previous findings of positive selection targeting this gene in East Asian populations only [10, 40].

Indeed, images such as Fig. 1 harbor information about processes such as population structure (changes in color gradients across populations) and adaptation (larger areas of the same color for populations targeted by positive selection) without being explicit about the phenomena that generated these signals. This is even more evident when investigating images of individual populations targeted by selection (Fig. 1c-e), and these are the ones which are currently used by ImaGene to quantify positive selection.

### Assessment of pipeline under various data and learning configurations

Herein, our aim is to evaluate the accuracy of detecting and quantifying a positive selective event under different settings of learning and data manipulation using ImaGene. We analyze data from one population only with diallelic polymorphisms with unknown ancestral state. Therefore, the corresponding images are the ones illustrated in Fig. 1e.

#### Manipulating images by sorting rows and columns improves detection

**Fig. 2:**
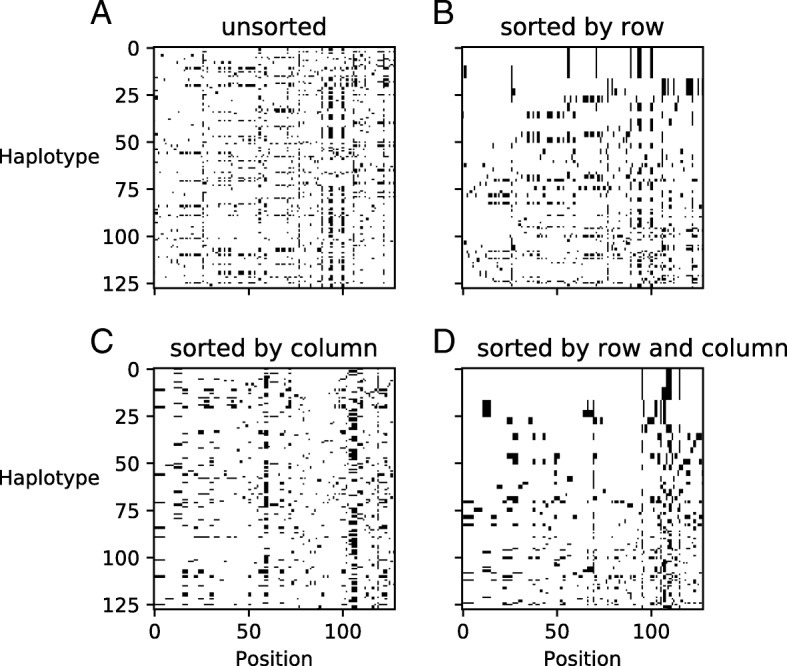
Image representations with different sorting conditions. The same image of genomic data is presented before (**a**) and after its rows (**b**), columns (**c**), or both (**d**) have been sorted by frequency of occurrence

In all images considered herein, each row represents a haplotype randomly sampled from the population. Therefore, any ordering of rows is purely stochastic and does not contain any viable information for our inferences (Fig. 2a). One possibility is to let the network learn this (lack of) feature. Alternatively, we can manipulate images by sorting rows according to certain criteria to help feature extraction. As positive selection, in the form of a selective sweep, creates a common haplotype with less frequent ones, previous studies either used a strategy of hierarchical sorting of rows by genetic distance [28] or modelled exchange-ability of haplotypes [30]. An additional possibility implemented in ImaGene is to enforce the abstract representation of images by sorting rows by their frequency of occurrence from top to bottom (Fig. 2b).

On the other hand, each column carries information about the relative position of polymorphisms along the locus. The ordering of columns contains information about linkage disequilibrium which can be informative for detecting selective sweeps [41]. However, this ordering is also affected by mutation and recombination events. Therefore, Imagene allows the generation of images by sorting columns by frequency from left to right (Fig. 2c) or by sorting both rows and columns by frequency (Fig. 2d).

We assessed whether the relative position of rows and/or columns carries more information than noise for detecting selection. Specifically, we calculated the accuracy of detecting positive selection against neutral evolution for different values of selection coefficient (200, 300, or 400 in 2*N*_*e*_ units with *N*_*e*_=10,000).

For this analysis, we implemented a CNN with three 2D convolutional layers of 32 units with kernel size of 3×3 and stride 1×1 each followed by a max-pooling layer with kernel size of 2×2. We finally applied a fully-connected layer with 64 units. We used ReLU (rectified linear unit) activation functions and a mini-batch size of 32. No zero-padding was applied. We removed columns corresponding to allele frequencies less than 0.01. After sorting, we resized all images to a dimension of 128×128 pixels.

To prevent overfitting, we used a “simulation-on-the-fly" approach where the algorithm is trained over newly generated data at each epoch. However, we retained the full training data set for ease of benchmarking. For each epoch, 10% for the training data was used as validation set while 10% of the whole data set was used for testing. A total of 50,000 simulations per class was generated.

**Fig. 3:**
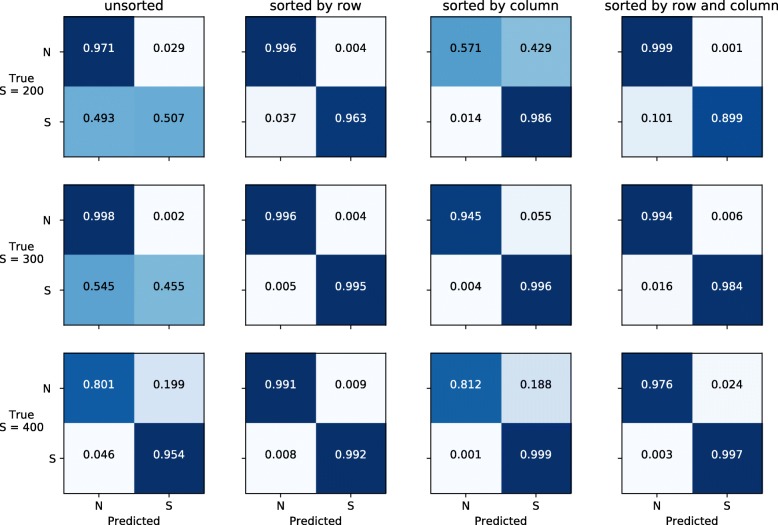
Accuracy of detecting positive selection using images with different sorting conditions. For each tested strength of positive selection (*S*={200,300,400}) we report the confusion matrices for predicting whether a genomic region is under neutrality (*N*) or selection (*S*) when images have been sorted with different conditions

Figure 3 shows the confusion matrices for the detection of positive selection under different sorting options (on the x-axis) and different values of the selection coefficient *S* (on the y-axis). Sorting rows by their frequency has a large impact in the performance and improves the prediction accuracy compared to using unsorted images especially for low values of the selection coefficient (Fig. 3, Additional file 1), in line with previous findings [28]. Notably, when rows and columns are both sorted, the accuracy is similar to the scenario of sorting rows only (Fig. 3). These results suggest that sorting both rows and columns can be a valuable option in case of unknown or uncertain mutation and/or recombination rates.

Furthermore, we noticed that inferences on double-sorted images do not require a final fully-connected layer in the CNN, as the spatial distribution of features is maintained. We tested this hypothesis and calculated the accuracy for prediction selection with *S*=300 without a final dense layer. We found a prediction accuracy of 0.9882 similar to what obtained when employing a final fully-connected layer (Additional file 1). Finally, we tested the prediction accuracy when adopting a larger kernel size 5×5 in the convolutional layers. We do not observe a significant change in accuracy under this condition (Additional file 1).

#### Quantification of natural selection is mildly robust to model assumptions

As the training data is generated by simulations conditional on a demographic model, the latter can have a notable effect on the prediction of natural selection. While the inference of parameters for demographic models is now achievable thanks to dramatic methodological advances [42–45], it less clear how to define a minimal configuration of size changes, especially for complex models with multiple populations.

We sought to test the robustness of our predictions to the underlying demographic model. Specifically, we assessed the prediction accuracy when training the network under a 3-epoch demographic model for a putative European human population [32], and testing it assuming a simpler 1-epoch model [32].

For this analysis, we implemented a CNN with three 2D convolutional layers of 32, 64 and 64 units, each followed by a max-pooling layer. Hyperparameters were set as previously described. No fully-connected layers were used. Images were resized to 128×128 pixels. We performed a multiclass classification for either neutral evolution or positive selection at different extent (*S*=200 or *S*=400).

**Fig. 4:**
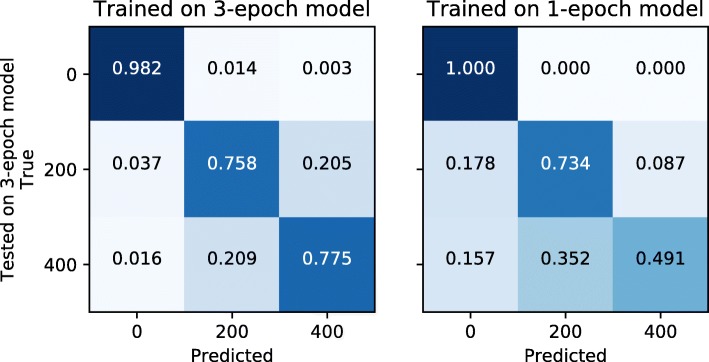
Accuracy of quantifying positive selection under different training models. We report the confusion matrices for predicting whether a genomic region is under neutrality (*S*=0), weak-to-moderate selection (*S*=200), or strong selection (*S*=400) when the network has been trained under the correct demographic model (3-epoch, on the left) or the incorrect one (1-epoch, on the right)

Figure 4 shows the accuracy in classifying events under three classes of either neutral or selective events when the network is trained with the same model used for testing (on the left) or a different one (on the right). While the detection of selection is not affected when the network is trained with a different demographic model, the accuracy for distinguishing between different extents of selection decreases (Fig. 4, Additional file 1). These results suggest that model misspecification during training has a larger effect for the quantification than for the prediction of natural selection.

### A quantification of natural selection from genomic data

After training, the CNN produces a posterior probability distribution for the parameter of interest, i.e. the selection coefficient. In fact, the output layer includes a *softmax* function that transforms the vector of class scores into probabilities. From this distribution, several statistical inferences can be made. ImaGene implements the estimation of continuous parameters using multiclass classification, by discretizing the parameter’s distribution into bins which are then considered as individual classes.

We sought to test the accuracy on estimating the selection coefficient by dividing the range of possible values (from 0 to 400) into 11 linearly spaced bins under different definitions of the true distribution: categorical, Guassian distribution centered around the true label with fixed standard deviation (0.5), or by randomly perturbing the true categorical distribution by a maximum step of 1 in either direction.

For this analysis, we implemented a CNN with three 2D convolutional layers of 32, 64 and 128 units, each followed by a max-pooling layer. Hyperparameters were set as previously described. Images were resized to 128×128 pixels. A total of 2,005,000 simulations were generated with selection coefficients drawn from a uniform prior distribution from 0 to 400. We then assigned each simulation to one of the 11 classes. We emphasize that here we did not attempt to optimize the architecture to minimize the bias in the estimation, but rather we aimed at comparing the accuracy under different configurations of the true parameter’s distribution in a multiclass classification task.

**Fig. 5:**
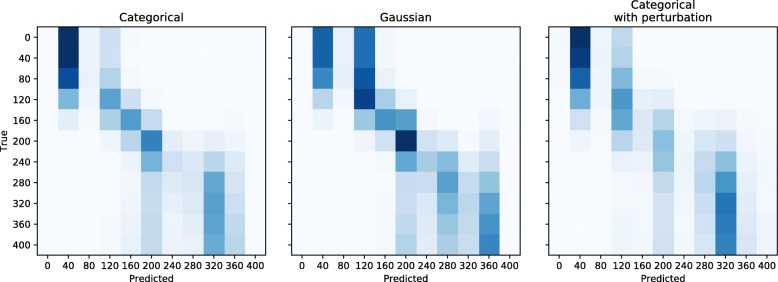
Accuracy of quantifying positive selection under different representation of the distribution of true labels. Confusion matrices for estimating selection coefficients into 11 intervals from 0 to 400. Classification was performed assuming a different representation of true labels, either as a categorical distribution, a Guassian distribution, or a perturbed categorical distribution

Confusion matrices between true and predicted labels (inferred as MAP values) show a general agreement among different methods to represent labels’ distribution (Fig. 5). The root mean squared error between true labels and estimated posterior means for the selection coefficient decreases by approx. 2% (corresponding to approx. 1 in 2*N*_*e*_ units) when using a Gaussian distribution instead of a categorical one. We did not observe an improvement in the estimation of the selection coefficient after randomly perturbing the true labels, possibly because of the limited number of discrete bins considered herein. However, using a perturbed categorical distribution for true labels leads to a lower standardized bias than the one obtained using a Gaussian distribution. The results suggest that incorporating uncertainty in the true labels may provide some advantages when estimating continuous variables with multiclass classification techniques.

**Fig. 6:**
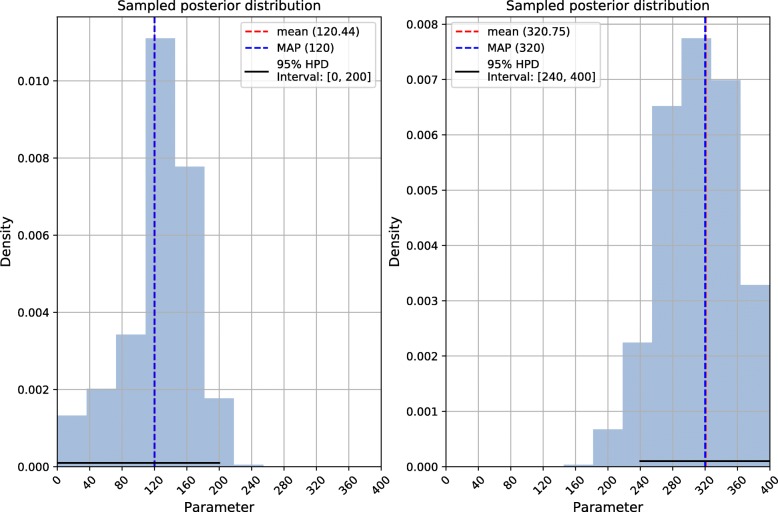
Sampled posterior distributions of selection coefficients. Histograms of 100,000 random samples from the posterior distributions of one case of weak-to-moderate selection (*S*=120, on the left) and one case of strong selection (*S*=320, on the right). Point estimates and credible intervals are reported

As an illustration, we provide the posterior probability distribution for selection coefficients under weak-to-moderate (*S*=120) and strong (*S*=320) selection for two cases where the estimation was accurate (Fig. 6). From the scores in the output layer, we calculated posterior mean and MAP values, as well as the HDPI (with *α*=0.05) after Monte Carlo sampling. Figure 6 shows that, for the case of weak-to-moderate selection (left panel), the HDPI is wide and includes the value of 0. However, the Bayes factor for testing a model with selection (coefficient larger than 0) vs. a model with no selection (coefficient equal to 0) is approx. 20, giving moderate support for the action of positive selection. Conversely, the Bayes factor in support of selection for the case of *S*=320 (right panel) is greater than 87,000, providing strong support towards positive selection occurring at this locus, as expected. ImaGene provides the full information on the probability distribution of the parameter of interest (e.g. the selection coefficient), allowing the user to derive several metrics and perform statistical tests.

## Discussion

In this study, we introduce a program, called ImaGene, for applying deep neural networks to population genomic data. In particular, we illustrated an application of convolutional neural networks to detect and quantify signatures of natural selection. We showed that ImaGene is flexible, scalable and fairly robust to data and model uncertainty.

In addition to these promising results, we foresee potential improvements and extensions to make its predictions more accurate and robust than the ones presented herein. Although there is currently no generalized formal framework for optimally designing a CNN for a particular classification problem, an extensive and systematic search over a wide range of architectures and hyperparameters is desirable to achieve maximum validation accuracy [46]. Furthermore, our choice of a random initialization method for setting the initial network parameters before training may be sub-optimal. Indeed, initializing the network with the parameters from a previously trained autoencoder has been shown to have a significantly positive impact on predictions [24].

It is important to assess how different loss functions can affect the estimation of continuous variables using multiclass classification. Also, while we evaluated several ways of manipulating labels after data discretization, further methods should be explored, including ordinal regressions [47] or the estimation of parameters (e.g. mean and standard deviation) of the posterior distribution [48].

The approach of resizing images on both axes has clear computational benefits. Resizing to a predefined square size allows for more efficient operations during the CNN optimization and for extended re-usability of the trained network in case of subsequent variations in sample size and genomic length. However, further investigations are in need to assess the effect of resizing input images, and on the trade-off between computational speed and accuracy when reducing their dimensionality.

In the current implementation, we do not use any spatial information on the distribution of polymorphisms, in contrast to other studies [28, 30]. While such information can improve prediction, here we show that even a purely abstract image representation of genomic data can be used for evolutionary inferences. Furthermore, using additional information on the physical distance between polymorphic sites may require a very detailed simulation of local genomic features (e.g. mutation rate, recombination rate, functionality) which is hardly achievable and may lead to loss of generality. Finally, it is not clear whether the use of color images showing the full information on nucleotidic content will increase prediction accuracy or simply slow the learning process. Nevertheless, further explorations of the potential of image representation of population genomic data are required.

Typically, CNNs are trained over a number of iterations (often called epochs), defined as one forward pass and one backwards pass over all the training data. When using this training method, data is re-seen by the learning algorithm multiple times. This often results in the overfitting of models, where CNN models learn specific images in the training data, along with any noise, rather than patterns important for classification. For limited training data and multiple epochs, regularization and dropout techniques are used to circumvent the issue of overfitting [49]. When training CNNs using simulated data, the amount of training data is only limited by computational time and space. “Simulation-on-the-fly" uses this ability to generate almost unlimited training data to prevent overfitting, as it involves carrying out simulations alongside training, so each data point is only seen once during training. This continuous simulation of data is carried out for many training iterations, until validation loss is sufficiently small, thus reducing overfitting [30]. Whilst effective, “simulation-on-the-fly” does not allow reproducible analyses for hyperparameter estimation [50]. ImaGene allows the user to choose a hybrid approach, where each iteration is performed over a fraction of the training data, and thus is visited by the CNN only once at the cost of producing a large training data at the beginning of the analysis.

Our current pipeline is integrated with *msms* [31], a commonly used program for simulating genomic data under selective scenarios. However, as ImaGene processes simulations in *ms* format, our pipeline is easily integrable with other programs such as *msprime* [51] and *SLiM* [52]. As the current time bottleneck in our pipeline is the generation and processing of *ms* files, we foresee the future opportunity of greatly improving computational efficiency by using state-of-the-art data representation of genealogical history of genomes in forward-time simulations [53, 54]. The use of efficient forward-time simulations is particularly welcomed, as they allow the generation of more realistic genomic data that take into account the functional context of the locus to analyze.

We have shown that, as expected, CNN-based quantification of natural selection is sensitive to violations of the assumed demographic history. To make sensible predictions from population genomic data, robustness should be assessed by training one single CNN with data coming from many different demographic histories or by adding model uncertainty within individual simulations. Commonly used methods to detect selection achieve robustness over the misspecification of demographic models by normalizing the information in their summary statistics against background signatures at the whole-genome level [55]. In a similar fashion, CNN-based estimation can generate Bayes factors for models supporting positive selection for each locus, and such empirical distribution can be used to detect outliers as candidates for targets of positive selection [7].

Summary statistics that incorporate information on the derived allele or haplotype frequency have been shown to have great power to detect strong and recent positive selection events [56]. However, in many cases, it is difficult to assign ancestral and derived allelic states with sufficient certainty [57]. In these cases, polarizing alleles based on their frequency in major or minor states can be directly calculated from sequence data with confidence. We predict that CNN-based inferences should achieve greater accuracy and shorter learning time when employing data incorporating information about ancestral and derived allelic states.

Additional accuracy in quantifying positive selection can be gained by using images from multiple populations simultaneously, either by stacking them or encoding differential allele frequencies in individual pixels. Such approach will mimic current methods to detect selection based on population genetic differentiation [10, 58, 59]. Similarly, incorporating temporal information from ancient genomes is likely to improve the prediction accuracy [60]. Finally, we foresee the application of this pipeline for the quantification of other selection events, e.g. balancing selection [61] or soft sweeps [62].

While ImaGene has been developed for deep sequencing data, SNP-chip data or targeted sequencing (e.g. exome) can be valid inputs, as long as simulations for the training data incorporate any ascertainment scheme used [63]. Also, this pipeline assumes that the data is phased, and that individual haplotypes are known. While this is a fair assumption for the study of model species, it is a strict requirement for the analysis of non-model species or with limited sample sizes. However, we foresee the potential use of unphased genotypes as input to any CNN-based classification. Finally, we predict the usefulness of such methodology for localizing functional variants targeted by natural selection, a task which is still challenging in population genomics [64]. As such, we plan to provide any updated analyses or extensions of ImaGene on its dedicated repository.

## Conclusions

In this study we provide a scalable pipeline for training a CNN classifier to detect and quantify signatures of natural selection from genomic data. We show how the prediction accuracy is affected by data preprocessing and learning settings. Furthermore, we show that misspecification of the demographic model used for generating the training set can affect the quantification of natural selection.

This study opens novel research directions for the use of deep learning, in particular of CNNs, in population genomics and human genetics [65]. Findings from these efforts will help better predict how evolution has shaped human predisposition to diseases [66] and unveil novel association with complex disorders.

## Availability and requirements

**Project name:** ImaGene

**Project home page:** https://github.com/mfumagalli/ImaGene

**Operating system(s):** Platform independent

**Programming language:** Python

**Other requirements:** Keras

**License:** GNU GPL v3

## Additional file

Additional file 1 Prediction accuracy of all tests performed in this study. In the file Additional_file_1.csv (.csv table) we report the classification task (e.g. binary or multiclass) and any specific option on data processing or learning setting along with the prediction accuracy for each tested case. (CSV 1 kb)

## Data Availability

The datasets generated and analysed in this study, along with all scripts used, are available at https://github.com/mfumagalli/ImaGene under a GNU GPL v3 license.

## Abbreviations

ANN: Artificial neural network
CEU: Utah residents with Northern and Western European ancestry
CNN: Convolutional neural network
HDPI: Highest posterior density interval
MAP: Maximum *a posteriori*
ML: Machine learning
*N*_*e*_: Effective population size
ReLU: Rectified linear unit
